# Calcium Carbide as a Caustic in Surgery

**Published:** 1912-03-23

**Authors:** 


### CALCIUM CARBIDE AS A CAUSTIC IN SURGERY.

At the Belgian Royal Academy of Medicine, Dr. Léon Desguin drew attention to the use of calcium carbide as a mild surgical caustic. He claims that it has distinct advantages over other forms of caustics. These are: (1) That it does not attack healthy dry skin, but reserves its effects for moist diseased tissues and has a predilective affinity for those that are the most affected. (2) That it is only slightly painful, is hæmostatic, antiseptic, and non-toxic. It is specially useful in dealing with badly-looked-after malignant ulcers, recurrent cancers, and ulcers of a doubtful nature.

In using the drug he employs the following technique: The flattest and thinnest flakes of calcium carbide that can be found in ordinary commercial samples are collected in a saucer or in a capsule. The skin surrounding the wound to be treated is next thoroughly cleansed with soap and water, after which it is carefully dried. The flakes are then taken up one by one and laid in rough juxtaposition over the whole surface of the diseased part. The application is next fixed in place by a dressing that cannot slip out of position. For this dressing highly absorbent material only should be used. A few days later the dressing is removed and the *débris* of carbide and necrosed tissues washed away by copious irrigations of normal saline solution, such portions of the carbide as remain adherent being removed subsequently by gentle scraping. A second application of the caustic is then made after carefully drying the surrounding skin, but it is not necessary to make use of such an extensive application as at the first sitting. A dressing is applied as before. After the third application it is usually possible to determine the degree of improvement effected, and as a matter of fact if the wound is not malignant there will be seen already a good number of places in which the surface shows healthy granulations, and nearly always the circumference will present an edge where cicatrisation is commencing. When these results are not obtained the inference in nearly all cases is that the lesion is of a malignant type, and for that reason thicker flakes of carbide should be used, though with care, because of its elective action on badly affected tissues.

As to his results, the author is led to believe that in many cases they have been very satisfactory, and he is satisfied that the effects he has produced have been better than those obtained with any other chemical caustic. Not only have cancers, said to be inoperable, become in this way capable of removal afterwards, but in some instances have come to present all the appearances of a perfect cure. It is especially in cases of cancer of the cervix uteri that the best results have occurred. Although the tumour has recurred in the body of the uterus, the cervix has remained firmly cicatrised, there being neither hæmorrhage nor symptoms of infection. Had these women only been willing to submit to operation when the cervical condition had been successfully treated, a complete cure would in all probability have resulted. In a case of carcinoma of the skin of the head of a man of fifty-three, which was inoperable by the ordinary surgical methods, a cure resulted by the use of the carbide, and was maintained for four years or so, after which the patient was lost sight of. The author concluded his remarks by begging his colleagues to give his method a trial, for he was certain that they would become converts if they did so.

